# Beyond COVID-19: Designing Inclusive Public Health Surveillance by Including Wastewater Monitoring

**DOI:** 10.1089/heq.2022.0055

**Published:** 2023-06-15

**Authors:** Rochelle H. Holm, Na'Taki Osborne Jelks, Rebecca Schneider, Ted Smith

**Affiliations:** ^1^ Christina Lee Brown Envirome Institute, School of Medicine, University of Louisville, Louisville, Kentucky, USA.; ^2^ Environmental and Health Sciences Program, Spelman College, Atlanta, Georgia, USA.; ^3^ Houston Health Department, Houston, Texas, USA.

**Keywords:** COVID-19, health equity, sewer, sewershed, underserved communities, wastewater-based epidemiology

## Abstract

Wastewater-based epidemiology is a promising and expanding public health surveillance method. The current wastewater testing trajectory to monitor primarily at community wastewater treatment plants was necessitated by immediate needs of the pandemic. Going forward, specific consideration should be given to monitoring vulnerable and underserved communities to ensure inclusion and rapid response to public health threats. This is particularly important when clinical testing data are insufficient to characterize community virus levels and spread in specific locations. Now is a timely call to action for equitably protecting health in the United States, which can be guided with intentional and inclusive wastewater monitoring.

Low-income and communities of color have been disproportionally impacted by the coronavirus disease 2019 (COVID-19) pandemic.^1–3^ Wastewater-based epidemiology (WBE), as public health sampling from existing public sewer infrastructure, provides an opportunity for more inclusive public health community monitoring through anonymous and passive sampling of community-generated fecal matter. With 85% of the U.S. residents on a piped sewer connection,^4^ which can be passively sampled, we have the potential to detect upticks in COVID-19 and other pathogenic viruses and react quickly with public health interventions, even in communities with limited testing or access to care.

We will need a method to sample the 15% unsewered U.S. residents,^4^ for example, through sampling as part of regular emptying operations; in the meantime, we can examine our current sampling strategy focused on wastewater treatment plants to ensure it is inclusive and serves the most vulnerable. We have choices regarding the deployment of this public health capability. Data presented by the Centers for Disease Control and Prevention^5^ show that 99% of the WBE samples are currently collected at a treatment plant (152,601/154,877 records).

Although WBE is convenient, relying on the current trajectory focus of pooled samples at the largest community points of access (i.e., wastewater treatment plants) may not equitably serve the public or public health. More thought should be given to where to sample, as we build on what has been established to date.

A specific example of ensuring that we do not leave some members of the urban underserved community behind is evidenced by case data in Louisville, Kentucky, where, since August 2020, WBE has been used to track severe acute respiratory syndrome coronavirus 2 (SARS-CoV-2) weekly at 17 wastewater sample locations to capture the entire county, but with a sampling design to intentionally oversample in urban underserved communities.^6^

Our Shawnee Park site, with a $27,695 household income (USD mean within catchment areas of reported block group median values), 89% minority, was reported as the lowest zone for weekly clinical testing rate administered for the period of December 20, 2021 to January 17, 2022, during the Omicron wave, at 2% (maximum was 7% of the population) and our geographically adjacent 34th Street site, with a $27,446 household income and 29% minority reported testing ranging from 2% to 6% of the population. Yet, on the other side of the city, our Muddy Forks site with a $103,304 household income, 8% minority, had a rate of weekly clinical testing administered to 10% of the population.

Each of these three areas discharges sewer to a common, large, treatment plant. The evolving WBE system in Louisville was intentionally set up to ensure that data from a single large treatment plant did not mask the infection story of low-income neighborhoods and communities of color. One difficulty of scale is the current approach to WBE still leaves behind a portion of the population on septic tanks or lacking improved sanitation systems such as people without homes; but 97% of Louisville households are connected to the sewer system.

Another example is that since May 2020, the city of Houston, Texas, population 2.16 million, has also been conducting weekly surveillance of the SARS-CoV-2 concentration in wastewater at 39 wastewater treatment plants. Public health officials find the data from this established WBE system extremely valuable, using trends in space and time to prioritize public health interventions especially in more vulnerable communities with lower clinical testing rates. Houston found that although trends of viral load in the wastewater for the city as a whole and by wastewater treatment plants are highly useful, they are especially important for tracking the virus in vulnerable communities.

When looking at two indicators of vulnerability, income and percentage minority population,^7,8^ Houston's ZIP codes in the lower decile by income and the highest decile by percentage of minority residents have statistically lower rates of clinical testing (*p*<0.02 and *p*<0.001, respectively) and higher positivity rates (*p*<0.001 and *p*<0.06, respectively) than ZIP codes in the opposite decile. Wastewater surveillance in sewersheds covering these vulnerable ZIP codes, where clinical testing is low and clinical cases are high, provides a consistent unbiased estimate of viral load.

For example, Houston's White Oak wastewater treatment plant has the lowest clinical testing rate of 39 sewersheds in Houston and covers a ZIP code in the top quartile of clinical positivity rate. As such, wastewater concentration trends are more helpful than the clinical testing rate in monitoring COVID-19 trends in that community.

Houston's successfully established WBE system could still be further improved by collecting more granular information, such as conducted within Louisville, within some wastewater treatment plants to better monitor conditions in underserved communities. Houston wastewater treatment plants vary in size, serving populations ranging from 10,000 to 500,000 people, and representing between 1 and 14 ZIP codes. ZIP codes using the largest wastewater treatment plant ranged in percentage minority population from 36% to 97.5%, in income percentile from the lowest to top 4, in positivity rate percentile from the 9th worst to 99th percentile, and in testing rate percentile from the highest to the 72nd lowest.

Subdividing these diverse communities out from a wastewater treatment plant would be helpful. But, there is also an issue of scale, where public health boundaries are often a zip code only domain unconnected to existing neighborhood sewer pipes.

Criticisms do not withstand WBE and often center on the use of law enforcement agencies that have access to drug use data by geographic boundaries, or where the population size of a WBE sample monitoring parameter becomes small enough to identify a contributing individual.^9^ In the context of COVID-19 and other infectious diseases, concerns about the potential use of wastewater testing data to stigmatize or restrict the movement and actions of highly affected and particularly that of vulnerable communities can be addressed as part of a formal policy implementing WBE for the greatest public health good. The success of such a framework begins by ensuring an equitable representation of underserved communities in the sampling design.

Data and policy solutions supporting inclusive counting of low-income and communities of color in the COVID-19 pandemic have largely ignored the equity in passive and anonymous community monitoring using existing city-wide wastewater infrastructure systems. Our experiences in Louisville and Houston are presented here as lesson learning to implement equity. We have learned equity and inclusion means, in part, being intentional about representative monitoring in urban underserved communities. The benefit of bringing rural communities into this equation should also not be overlooked.

The wastewater testing trajectory on monitoring primarily at treatment plants leaves portions of the community diluted and out of existence in some neighborhoods or invisible in exurban and rural settings. There is an opportunity, however, for this approach to become a powerful tool to help advance health equity. This is particularly important when clinical testing data are insufficient to characterize the level of community virus or to trigger the deployment of resources to improve public health outcomes to the areas that most need investment. Now is a timely call to action for equitably protecting health in the United States, which can be guided with intentional and inclusive wastewater monitoring.

## Abbreviations Used

COVID-19: coronavirus disease 2019
SARS-CoV-2: severe acute respiratory syndrome coronavirus 2
WBE: wastewater-based epidemiology

## References

[1] Bilal U, Tabb LP, Barber S, et al. Spatial inequities in COVID-19 testing, positivity, confirmed cases, and mortality in 3 US cities: An Ecological Study. Ann Intern Med 2021;174(7):936–944; doi: 10.7326/M20-3936 33780289 PMC8029592

[2] Tai DBG, Shah A, Doubeni CA, et al. The disproportionate impact of COVID-19 on racial and ethnic minorities in the United States. Clin Infect Dis 2021;72(4):703–706; doi: 10.1093/cid/ciaa815 32562416 PMC7337626

[3] Shiels MS, Haque AT, Haozous EA, et al. Racial and ethnic disparities in excess deaths during the COVID-19 pandemic, March to December 2020. Ann Intern Med 2021;174(12):1693–1699; doi: 10.7326/M21-2134 34606321 PMC8489677

[4] World Health Organization and United Nations Children's Fund (UNICEF). Progress on household drinking water, sanitation and hygiene 2000–2020: Five years into the SDGs. Geneva; 2021. Available from: https://data.unicef.org/resources/progress-on-household-drinking-water-sanitation-and-hygiene-2000-2020/ [Last accessed: December 12, 2021].

[5] Centers for Disease Control and Prevention. SARS-CoV-2 RNA Levels in Wastewater in the United States. Available from: https://covid.cdc.gov/covid-data-tracker/#wastewater-surveillance [Last accessed: March 23, 2022].

[6] Yeager R, Holm RH, Saurabh K, et al. Wastewater sample site selection to estimate geographically-resolved community prevalence of COVID-19: A sampling protocol perspective. GeoHealth 2021;5(7):e2021GH000420; doi: 10.1029/2021GH000420 PMC8240399 34222738

[7] Houston State of Health: Informing Action with Health Data. Median Household Income, Measurement Period: 2015–2019. Available from: https://www.houstonstateofhealth.com/indicators/index/view?indicatorId=315&localeId=38539 [Last accessed: March 29, 2022].

[8] United States Census Bureau. American Community Survey 5-year 2019. Available from: https://www.census.gov/data/developers/data-sets/acs-5year.html [Last accessed: March 29, 2022].

[9] Hrudey SE, Silva DS, Shelley J, et al. Ethics guidance for environmental scientists engaged in surveillance of wastewater for SARS-CoV-2. Environ Sci Technol 2021;55(13):8484–8491; doi: 10.1021/acs.est.1c00308 34101444

